# The Impact of Pain Invisibility on Patient-Centered Care and Empathetic Attitude in Chronic Pain Management

**DOI:** 10.1155/2018/6375713

**Published:** 2018-09-24

**Authors:** Emilie Paul-Savoie, Patricia Bourgault, Stéphane Potvin, Emilie Gosselin, Sylvie Lafrenaye

**Affiliations:** ^1^School of Nursing, Faculty of Medicine and Health Sciences, Université de Sherbrooke, Sherbrooke, QC, Canada; ^2^Centre de Recherche de L'Institut Universitaire en Santé Mentale de Montréal, Department of Psychiatry, Faculty of Medicine, Université de Montréal, Montreal, QC, Canada; ^3^Department of Pediatrics, Faculty of Medicine and Health Sciences, Université de Sherbrooke, Sherbrooke, QC, Canada

## Abstract

**Objectives:**

The use of interdisciplinary patient-centered care (PCC) and empathetic behaviour seems to be a promising avenue to address chronic pain management, but their use in this context seems to be suboptimal. Several patient factors can influence the use of PCC and empathy, but little is known about the impact of pain visibility on these behaviours. The objective of this study was to investigate the influence of visible physical signs on caregiver's patient-centered and empathetic behaviours in chronic pain context.

**Methods:**

A convenience sample of 21 nurses and 21 physicians participated in a descriptive study. PCC and empathy were evaluated from self-assessment and observer's assessment using a video of real patients with chronic pain.

**Results:**

The results show that caregivers have demonstrated an intraindividual variability: PCC and empathetic behaviours of the participants were significantly higher for patients who have visible signs of pain (rheumatoid arthritis and complex regional pain syndrome) than for those who have no visible signs (Ehler–Danlos syndrome and fibromyalgia) (*p* < 0.001). Participants who show a greater difference in their patient-centered behaviour according to pain visibility have less clinical experience.

**Discussion:**

The pain visibility in chronic pain patients is an important factor contributing to an increased use of PCC and empathy by nurses and physicians, and clinical experience can influence their behaviours. Thus, pain invisibility can be a barrier to quality of care, and these findings reinforce the relevance to educating caregivers to these unconscious biases on their behaviour toward chronic pain patients.

## 1. Introduction

Chronic pain is a common public global health problem, associated with significant disability and many social consequences [1]. Chronic pain affects people of all ages, with a particularly high prevalence in adults, ranging from 10 to 55% [2]. People with chronic pain are known to consult health professional frequently, leading in a heavy economic burden [3, 4]. To be effective, the treatment of chronic pain must consider biological, psychological, and social factors simultaneously [5]. Thus, the use of interdisciplinary patient-centered care (PCC) seems to be a promising avenue to address chronic pain management [6, 7].

There are many definitions of PCC in the context of nursing and medicine, but four dimensions are common to most cited definitions: patient-as-person, biopsychosocial perspective, sharing power and responsibility, and therapeutic alliance [8–10]. The use of this approach is related with many clinical benefits for patients with chronic pain [7, 11–13]. More specifically, researchers have shown that PCC resulted in a decrease in the number of tender points and psychological distress in fibromyalgia patients [11]. Qualitative findings support that PCC allows nurses to improve their assessment and to provide better anticipatory guidance and coaching [12].

In addition, many researchers suggest that empathy is necessary for the use of PCC [8, 14, 15]. In the model of Bilodeau et al., “having an empathetic presence” is an important dimension of PCC [8]. In healthcare, empathy is defined as a cognitive attribute involving an understanding of the patient's experience and perspective, as a separate individual, combined with an ability to communicate that understanding to the patient [16]. Interestingly, it has been suggested that high levels of empathy were related to positive outcomes for patients [17–19] and especially with chronic pain patients [20].

Although many studies support the benefits of PCC, [21]its use in chronic pain management seems to be challenging and suboptimal [20, 22]. Patient-centered behaviour or communication does not necessarily translate into a “unique recipe,” and caregivers seem to use a flexible style according to patient characteristics [23]. Over the last years, it has been suggested that several patient factors can influence the use of PCC, such as age, gender, and level of education [24–26]. For example, caregivers would tend to use more PCC behaviour with women, older, and nonsmoker patients [24, 26]. Moreover, some authors have shown that physicians tend to use more PCC when patients reported more physical symptoms, such as nausea, dry mouth, or constipation, and when they rated patients' health condition as more severe [26]. However, in chronic pain context, little is known about the impact of the “visibility” of physical signs on patient-centered behaviour.

Patient factors may also influence caregivers' empathy. Indeed, both behavioral and functional neuroimaging measures have demonstrated that some stigma or prejudice could modulate empathy [27]. Since many patients with chronic pain do not display any visible physical symptoms and remain stoic when they feel pain [28], it is important to assess the impact of this specific factor on patient-centered care and empathetic behaviour.

Various methods have been used for measuring the use of PCC and empathy, such as self-rating and observer rating [9, 23, 29]. Self-assessment instruments are the most common strategy used for measuring PCC and empathy, but they do not consider the influence of different patient factors such as the presence or absence of visible physical signs. The observation of PCC and empathy in real clinical encounters may raise some ethical and methodological issues, including the inability to have standardized visits, which introduces confounding variables. The use of standardized patient simulations is a very expensive strategy [30]. Videos of real patients with chronic pain could overcome these limitations by allowing a standardized and repetitive assessment of attitudes and behaviours of caregivers [31].

Thus, the main objective of this study was to investigate the influence of the presence of visible physical signs on patient-centered and empathetic behaviours of nurses and physicians using videos of real patients suffering from chronic pain. In view of the limited knowledge on the topic, we also sought to identify, in an exploratory fashion, the characteristics of the caregivers who respond differently to the presence or absence of visible signs of pain.

## 2. Materials and Methods

### 2.1. Study Design and Settings

This study is part of a larger study which investigated PCC and empathy in chronic pain management [32]. A descriptive design was used, conducted from May to November 2013, in the province of Quebec, Canada. The Scientific and Human Ethics Committee of the institution where the study took place approved the research protocol. A population composed of nurses and physicians has been targeted since interdisciplinary pain management is recommended [8]. A convenience sampling approach was chosen, and participants were recruited through advertisements and referrals. Participants were recruited from 16 different healthcare centres, including urban, semiurban, and rural centres, in the province of Quebec, Canada.

### 2.2. Participants

All participants gave their written, informed consent, and a coding system was used to keep the data confidential. To take part in the study, the participants need to (i) be active members of the Quebec Board of Nurses or the Quebec College of Physicians, (ii) have chronic pain patients in their routine practice, and (iii) speak French. After signing the informed consent, a sample of 21 nurses and 21 physicians took part in the study. A minimum of 38 participants was needed to detect a moderate difference (*d* = 0.5) between visible and invisible pain conditions with a power of 85% and a type-1 error of 5%. Participants did not know the detailed purpose of the study to avoid social desirability bias, but they were informed that pain management was investigated.

### 2.3. Self-Assessment Measures

The practice orientation of each participant was measured with the French version of the Patient Practitioner Orientation Scale (F-PPOS) [33]. This self-administrated questionnaire is designed for the assessment of practitioners' or future-practitioners' attitudes and orientations in their care approach. This scale contains 18 items divided into two subscales: “sharing” and “caring,” and the four dimensions of PCC are represented. For each item, the caregiver is asked to indicate his or her level of agreement on a 6-point Likert scale (strongly agree to strongly disagree). A total score, ranging from patient-centered (a score of 6.00) to disease-centered (a score of 1.00), can be calculated with the addition of the two subscales. The original version of the PPOS has good face validity and acceptable internal consistency for the total scale (an alpha of 0.89) [34]. The French version of the PPOS has good content validity and acceptable internal consistency for the total scale an (alpha of 0.60) [34], since the minimum threshold is 0.50 [35].

The self-rated empathy was measured with the French version of the Jefferson Scale of Physician Empathy (F-JSPE) (P. Bourgault, S. Lavoie, and M. Grégoire et al., unpublished data, June 2009). This self-administrated questionnaire comprises 20 items divided into 4 dimensions: (i) adopting the patient's perspective, (ii) understanding the patient's experiences, feelings, and signals, (iii) ignoring the patient's perspective, and (iv) adopting the patient's way of thinking. The F-JSPE also includes a single item measuring the value placed on empathy. Participants respond to items on a 7-Likert scale. The total score, ranging from not empathic (a score of 20) to empathic (score of 140), can be calculated with the addition of all the items. The original version of the JSPE has good criterion-related validity with the Empathic Concern Scale of the Interpersonal Reactivity Index (*r* = 0.40), internal consistency (an alpha of 0.87 to 0.89), test-retest reliability (test-retest reliability coefficient = 0.65), and construct validity [16, 36]. The F-JSPE has good content validity and acceptable internal consistency (an alpha of 0.77) (P. Bourgault, S. Lavoie, and M. Grégoire et al., unpublished data, June 2009).

### 2.4. Observers' Assessment Measures

The PCC behaviour of each participant was measured with the Sherbrooke Observation Scale of patient-centered care (SOS-PCC) [31]. This instrument has been developed for the assessment of PCC behaviour of a professional caregiver by a trained observer, in the experimental clinical setting. Nine items divided into 4 dimensions (patient-as-person, biopsychosocial perspective, sharing power and responsibility, and therapeutic alliance) are measured on a 4-points Likert scale. The instrument was originally developed in French for a population of nurses and physicians. A total score, ranging from disease-centered (a score of 9) to patient-centered (a score of 36), can be calculated by the addition of all items. This instrument has good content validity, internal consistency (an alpha of 0.88), and inter-rater reliability (an intraclass coefficient (ICC) of 0.93) [31].

An adapted French version of Reynolds Empathy Scale (F-RES) has been used for the assessment of empathetic behaviour by a trained observer [37]. The F-RES consists of 9 items with a categorical rating scale (“yes,” “no,” or “incomplete”). This instrument has good internal consistency (an alpha of 0.70) and inter-rater reliability (an ICC of 0.85) [38]. In this study, we used a 7-item version with a 4-point Likert scale to make it more responsive to the context of videos. A total score, ranging from not empathetic (a score of 7) to empathetic (a score of 28), can be calculated by adding all items.

### 2.5. Procedure

**Figure 1:**
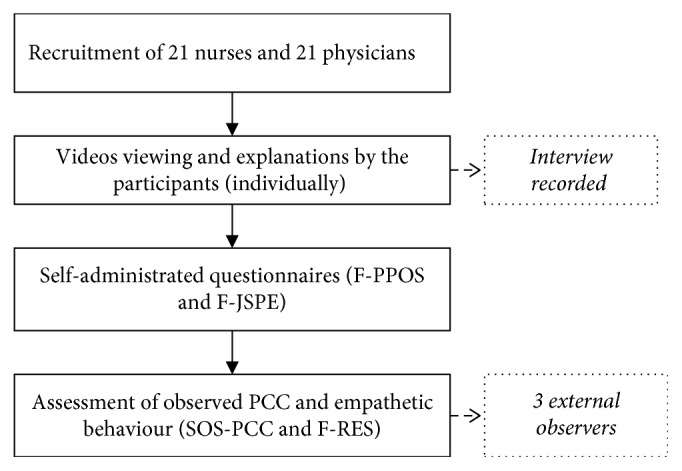
Conduct of the study. F-PPOS indicates the French version of the Patient-Practitioner Orientation Scale, F-JSPE indicates the French version of the Jefferson Scale of Physician Empathy, PCC indicates patient-centered care, SOS-PCC indicates Sherbrooke Observation Scale of patient-centered care, and F-RES indicates the French version of the Reynolds Empathy Scale.

All study participants watched four videos of real patients with chronic pain and were subsequently interviewed individually. These 4-minute videos showed female patients aged between 16 and 45 years-old, with different pathologies in which chronic pain was experienced (rheumatoid arthritis, complex regional pain syndrome (CRPS), Ehlers–Danlos syndrome, and fibromyalgia). A more detailed description of the development and content of these videos is available elsewhere [31]. In these videos, some patients had no visible physical signs (Ehlers–Danlos syndrome and fibromyalgia) and others had obvious visible deformities in the upper limb (rheumatoid arthritis and CRPS). After viewing the videos, participants had to describe the management plan that they would provide for each patient, and these answers were video recorded. At the end of the session, each participant responded to the self-administrated questionnaires. After the data collection (*n*=42 participants), three external observers watched the recorded interviews of each participant (4 interviews/participant). The group of observers consisted of a resident in psychiatry, a nurse, and a PhD student in the healthcare field. The observers were selected based on their experience in the healthcare field (more than five years) and their complementary expertise (medicine, nursing, and research). To ensure the standardization in their assessment, they had previously been trained by the research team to complete the observation scale. They evaluated the PCC and empathetic behaviour demonstrated by participants for each video using the SOS-PCC and the F-RES.  Figure 1 showed the conduct of the study.

### 2.6. Data Analysis

Statistical analyzes were performed using the software SPSS version 18.0. To describe continuous variables, mean (standard deviation) was used, whereas frequency (percentage) was used for nominal and categorical variables. To compare patient-centered and empathetic behaviour of the participants (*n*=42) according to the presence or absence of visible physical signs in the patients presented in the videos, paired *T*-tests were used on the mean score of the three observers.

To investigate if the clinical experience can influence the modification of the behaviours according to the pain visibility, for each study participant, we calculated the difference of SOS-PCC and F-RES between the his/her behaviour for the patients with visible signs and the patients without visible signs. We divided the participants into two groups according to these differences: the participants who rated the two groups of patients similarly and those who rated the two groups of patients differently. To determine whether a participant showed similar or different behaviours, we have used the mean scores of SOS-PCC and F-RES for the thresholds. We used paired *T*-tests to compare the differences in clinical experience between the two groups study participants. The statistical level of significance was set at *p* < 0.05. We do not have any missing data.

## 3. Results and Discussion

### 3.1. Participants' Characteristics

The sample included 42 native French-speaking caregivers ranging from 27 to 67 years (*M* = 46.12 years; SD = 10.84) and the majority was women (69%). Twenty-one (50%) of the participants were nurses, and 21 (50%) were physicians. Our sample was composed of nurses working in different settings, general practitioners, and medical specialists (physiatrist, rheumatologist, orthopaedist, radiologist, nephrologist, neurologist, and psychiatrist). The nurses and physicians had an average of 19.74 years of clinical experience. In this group, participants self-reported a mean overall orientation for PPC of 4.82. More specifically, the mean was 4.49 for the sharing subscale and 5.16 for the caring subscale. The participants self-reported a mean of 116.53 for empathy. These results suggest moderate levels of PCC and empathy when participants assess themselves. For the observer's assessment with observation scales, the results are presented for each video. For observed PCC, the mean for the 4 videos was 25.94, and for observed empathy, the mean was 20.70.  Table 1 shows the characteristics of these participants.

### 3.2. Patient-Centered and Empathetic Behaviour according to the Presence or Absence of Visible Physical Signs

**Figure 2:**
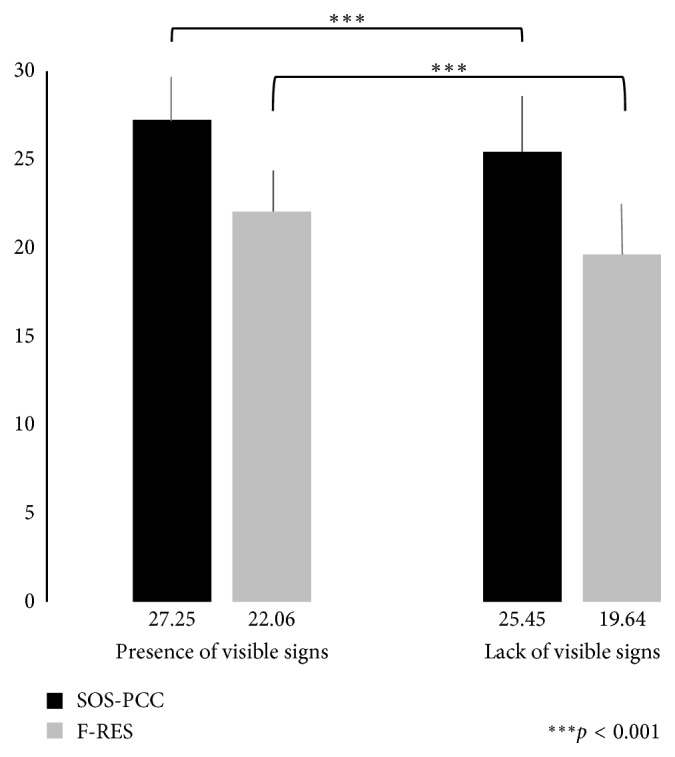
Influence of pain visibility on patient-centered care and empathy. Patient-centered care and empathy behaviours were observed by external raters. SOS-PCC indicates Sherbrooke Orientation Scale of patient-centered care, and F-RES indicates French version of the Reynolds Empathy Scale.

In total, 168 interviews in response to the videos were videotaped successfully. We divided these interviews in two groups: (i) interviews in response to the videos presenting patients with visible physical signs (rheumatoid arthritis and CRPS) and (ii) interviews in response to the videos presenting patients without visible physical signs (Ehlers–Danlos syndrome and fibromyalgia). Regarding the observed patient-centered and empathetic behaviours, the mean for SOS-PCC and F-RES was calculated for both groups. The results support that patient-centered and empathetic behaviour was significantly higher for the group of patients with visible physical signs (*p* < 0.001) (Figure 2). The group of participants who show a greater difference in their patient-centered behaviour according to pain visibility have less clinical experience than the group that had similar behaviour for both patients with and without physical visible signs (*p*=0.03). For empathetic behaviour, the difference is not statistically significant (*p*=0.23) (Table 2).

## 4. Discussion

The results suggest that our sample of caregivers have moderate levels of self-reported PCC and empathy. In comparison with other studies, nurses and physicians who have participated in our study used more PCC than other groups of nurses and physicians with similar sociodemographic characteristics [38, 39]. For self-reported empathy, our sample was comparable to other nurses and physicians [40].

The main purpose of this descriptive study was to investigate the influence of the presence or absence of visible physical signs on caregiver behaviours and attitudes in chronic pain management. PCC and empathy were assessed from two perspectives, using a combination of self-administrated questionnaires and observation scales using innovative videos of real patients with chronic pain. Our results show that nurses and physicians show intraindividual variability. Indeed, we found that PCC and empathetic behaviour of nurses and physicians vary according to the presence or absence of visible physical signs in patients with chronic pain.

Interestingly, another research team have demonstrated that residents in internal medicine have displayed more patient-centered behaviour when they consider that the patient has a more severe health condition and more visible physical symptoms [26]. In the same vein, another research group has demonstrated that patient-centered practice style of physicians was positively related with higher patient self-reported physical health status [24]. Although the severity of the disease is not automatically associated with the presence of visible physical symptoms, the results of clinical observations have shown that patients with more severe diseases were given longer consultations and the opportunity to talk more about their medical condition [41]. These observations have a great importance in the context of chronic pain, since these patients often have no visible physical signs, and can remain stoic when they feel pain [28] because their condition is often present for several years. For example, patients with neuropathic pain suffer from allodynia and hyperalgesia, but they often have no obvious physical signs. Moreover, some authors have suggested that professional caregivers were baffled by the lack of correlation between pain intensity verbally and nonverbally expressed by chronic pain patients and their medical condition [42].

It has been shown that physicians tend to rely on their initial assessment of the pain of their patients with chronic pain even when this initial assessment underestimates the pain subsequently reported verbally by the patients [43]. Thus, if chronic pain patients are not asked about their preoccupations and their medical condition's severity, stoic patients and those with less visible physical signs are more likely to receive suboptimal personalized care, with a more disease-centered orientation and less empathetic behaviour. These findings suggest that nurses and physicians adjust their practice orientation and empathy according to the clinical condition. It could explain why many patients with chronic pain are frustrated after their clinical encounters. This has a particular impact since these interactions are prominent in their experience [43].

Interestingly, we found that the clinical experience can influence the behaviours of nurses and physicians. Indeed, caregivers with more years of experience are less likely to change their behaviour were according to the presence or absence of physical visible signs. Many studies have investigated the influence of clinical experience on PCC and empathy [44, 45], but little is known about how clinical experience could modulate these variables. A brain imaging study showed that clinical exposure could reduce empathy for pain [46]. More specifically, the authors investigated cortical activity among physicians and control subjects exposed to a series of visual stimuli with body parts in painful and painless situations. Their results indicated that the cortical structures associated with empathy for pain were significantly activated in the control subjects, but not in physicians. It is suggested that these observations are the consequence of a protective regulatory mechanism in people who work daily with pain in order to prevent their distress [46]. Thus, it is possible to believe that the behaviours of the most experienced caregivers are less influenced by the presence of visible signs of pain. However, our results show no difference in PCC and empathy levels according to the clinical experience (*p* > 0.05). It is also possible that the most experienced caregivers are more aware of the impact of pain visibility, and they consider this potential bias in their interventions.

One potential limitation of our study is the lack of a real interaction between participants and patients in the videos. As a result of its methodological advantages and low cost, we used standardized videos of real chronic pain patients. However, the observation scales used for the assessment of PCC and empathetic behaviours were adapted for this kind of situation. Another potential limitation is the reactivity bias. It is possible that participants have positively modified their answers regarding the treatment plan that they would provide in reality. However, many efforts have been made to mitigate this potential bias. First, the participants did not know the variables under study at the time of data collection. Secondly, the questionnaires were distributed at the end of the experimental session in order to avoid a modification in behaviour, conscious or not, from participants.

## 5. Conclusions

In the last years, several studies have shown that practice orientation and empathy of professional caregivers could be influenced by many patients' factors [43, 45, 46], and our results are consistent with these previous findings. To our knowledge, this is the first study showing a direct influence of visible physical signs on PCC and empathy in the context of chronic pain management. Those observations are to be considered, since chronic pain patients often do not show any apparent physical signs. Thus, these patients are more likely to receive suboptimal pain management, more disease centered and devoid of empathic behaviour. This raises the importance of educating caregivers and future caregivers to these unconscious biases and the potential impact on chronic pain patients. The impact of the presence or absence of apparent physical signs must also be considered for research in chronic pain since it is a potential confounding factor. Future studies could utilize real clinical encounters and other populations to replicate these results.

## Figures and Tables

**Table 1:** Results of self-administrated questionnaires.

	Participants (*n*=42)
Age, mean (SD)	46.12 (10.84)
Gender, *n* (%)	
Male	13 (31)
Female	29 (69)
Profession, *n* (%)	
Nurse	21 (50)
Physician	21 (50)
Clinical experience, mean (SD)	19.74 (10.34)
F-PPOS total, mean (SD)	4.82 (0.39)
Sharing, mean (SD)	4.49 (0.59)
Caring, mean (SD)	5.16 (0.37)
F-JSPE, mean (SD)	116.53 (9.80)
SOS-PCC, mean (SD)	25.94 (3.86)
Patient with rheumatoid arthritis, mean (SD)	27.12 (3.88)
Patient with CRPS, mean (SD)	27.38 (4.30)
Patient with Ehlers–Danlos syndrome, mean (SD)	26.02 (4.38)
Patient with fibromyalgia, mean (SD)	24.87 (5.11)
F-RES, mean (SD)	20.70 (3.41)
Patient with rheumatoid arthritis, mean (SD)	21.89 (3.59)
Patient with CRPS, mean (SD)	22.30 (3.25)
Patient with Ehlers–Danlos syndrome, mean (SD)	20.34 (3.81)
Patient with fibromyalgia, mean (SD)	18.94 (4.05)

SD = standard deviation; F-PPOS = French version of Patient-Practitioner Orientation Scale; F-JSPE = French version of Jefferson Scale of Physician Empathy; SOS-PCC = Sherbrooke Orientation Scale of patient-centered care; CRPS = complex regional pain syndrome; F-RES = French version of Reynolds Empathy Scale.

**Table 2:** Influence of clinical experience on caregivers' behaviours in the presence or absence of physical visible signs.

	Similar ratings	Different ratings	*p* value
Patient-centered behaviour			
*n*	14	28	
Clinical experience, years (SD)	24.64 (8.68)	17.29 (10.36)	0.03
Empathetic behaviour			
*n*	20	22	
Clinical experience, years (SD)	20.35 (9.73)	19.18 (11.05)	0.23

SD = standard deviation.

## Data Availability

The data used to support the findings of this study are available from the corresponding author upon request.
